# Cation functional group effect on SO_2_ absorption in amino acid ionic liquids

**DOI:** 10.3389/fchem.2023.1113394

**Published:** 2023-02-02

**Authors:** Hasan Siami, Mohammad Razmkhah, Fatemeh Moosavi

**Affiliations:** ^1^ Department of Chemistry, Ferdowsi University of Mashhad, Mashhad, Iran; ^2^ Salim Green Health R&D, Ferdowsi University of Mashhad, Mashhad, Iran

**Keywords:** absorption, amino acid ionic liquid, density functional theory, sulfur dioxide, functionalized cation

## Abstract

**Introduction:** The effect of the functional group of the cation on SO2 acidic gas absorption by some designed amino acid ionic liquids (AAILs) was studied.

**Methods:** An isolated pair of glycinate anion and pristine imidazolium-based cation, as well as decorated cation functionalized by hydroxyl (OH), amine (NH_2_), carboxylic acid (COOH), methoxy (OCH_3_), and acetate (CH_3_COO) groups, were structurally optimized by density functional theory (DFT) using split-valence triple-zeta Pople basis set.

**Results and Discussion:** The binding and Gibbs free energy (ΔG_int_) values of SO_2_ absorption show the AAIL functionalized by the COOH group is the most thermodynamically favorable green solvent and this functional group experiences the closest distance between anion and captured SO_2_ and *vice versa* in the case of cation … SO_2_ which may be the main reason for being the best absorbent; in addition, the highest net charge-transfer amount of SO_2_ is observed. Comparing the non-covalent interaction of the systems demonstrates that the strongest hydrogen bond between captured gas and anion, as well as π-hole, and van der Waals (vdW) interaction play critical roles in gas absorption; besides, the COOH functional group decreases the steric effect while the CH_3_COO functional group significantly increases steric effect after absorption that declines the hydrogen bond.

## Introduction

Looking at the environmental effects caused by sulfur dioxide (SO_2_) gas, effective technology for flue gas desulfurization (FDG) is of great practical importance. SO_2_, as an acidic gas, is released from the combustion of fossil fuels containing sulfur in power plants, incinerators, and boilers; the roasting of sulfide ore in metallurgy and sulfuric acid industry are the major sources of this atmospheric pollution as well as the main component of acid rain and fog. The high toxicity of this gas leads to many problems for human health and due to the presence of moisture in the atmosphere, the oxides in the air react with SO_2_ and produce sulfuric acid, which is the main precursor for acid rain (Cui et al., 2021a; Zhu et al., 2021; Mohammadi et al., 2022; Zhang et al., 2022). The emission of SO_2_ leads to the formation of acid rain, photochemical smog (Li et al., 2021a), and has adverse effects on the quality of the ecosystem. From the other side of view, SO_2_ is a stronger acid than carbon dioxide gas, and the presence of sulfur dioxide in small amounts in the flue gas has adverse effects on the removal of CO_2_ gas. Moreover, SO_2_ has an impact on the post-combustion CO_2_ capture applications and reduces the CO_2_ absorption capacity and lifetime of the absorbent as well as increasing the operating costs (Zhang et al., 2020). As a result, SO_2_ capturing is of great practical importance.

Conventional desulfurization methods, such as ammonia/amine scrubbing, wet washing, and limestone scrubbing, lead to the production of low-value by-products, solid gypsum waste, wastewater, highly polluted water, and volatile organic compounds (VOCs) caused by solvent evaporation (Jiang et al., 2020; Sheng et al., 2021). In other words, they create secondary pollution and have limited application due to low reversibility. A prerequisite for a successful gas capturing technology is low price and low energy consumption. SO_2_ gas reacts with amines and creates irreversible salts, accordingly it reduces the lifetime of the absorber and increases the operating cost. Therefore, effective, clean, and green technology should be explored.

Attributable to the properties of thermal stability, low vapor pressure, physical and chemical stability, adjustability, non-volatility, tuneability, and great tendency to capture SO_2_, ionic liquids (ILs) are a hot research topic in the field of gas absorption as green and clean solvents (Mondal and Balasubramanian, 2016; Yang et al., 2017; Cui et al., 2021b; Rashid, 2021; Liu et al., 2022a). In addition, functional ILs have shown efficient SO_2_ absorption (Ren et al., 2018; Wang et al., 2020; Geng et al., 2022). Doblinger et al. (2021) have experimentally measured polar gas SO_2_ solubility in non-functionalized IL, 1-ethyl-3-methylimidazolium bis(trifluoromethylsulfonyl)imide, and the functionalized alkyl side-chain of cation by hydroxyl, cyanide, and methyl benzyl. SO_2_ was physically dissolved in target ILs. To answer why functionalized ILs are applied for absorption one should state that typical ILs have physical absorption and therefore limited absorption capacity; adding functional groups such as hydroxyl, amine, and ether leads to more absorption of SO_2_ based on physical and chemical absorption (Liu et al., 2022b). ILs based on imidazolium (Li et al., 2021a; Doblinger et al., 2021), guanidinium (Wang et al., 2007; Geng et al., 2022), and pyridinium (Zeng et al., 2014; Yan et al., 2019) can physically dissolve the gas. In addition to experimental efforts (Huang et al., 2014; Cui et al., 2020; Wang et al., 2021a; Cui et al., 2021b; Geng et al., 2021; Liu et al., 2022b; Hou et al., 2022) and theoretical and computational studies (Wang et al., 2007; Wang et al., 2022a; Mohammadi et al., 2022) to investigate the solubility of gases in ILs, *ab initio* and first principle investigations have also been conducted to inspect the structure and mechanism of the complex of IL and SO_2_ gas (Gu et al., 2013; Herrera et al., 2017; Cui et al., 2020; Wang et al., 2020; Li et al., 2021a; Li et al., 2021b; Yin et al., 2021; Zhu et al., 2021; Liu et al., 2022b). For example, Wang et al. (2007) simulated the solubility of CO_2_ and SO_2_ in guanidium-based IL and Zhang et al. (2020) investigated the stability effect of ILs during the CO_2_ absorption process in the presence of SO_2_. Due to the ability of chemical removal of SO_2_ by ILs, the chemical reaction of SO_2_ gas has been taken into consideration through *ab initio* quantum computations (Piacentini et al., 2022).

ILs also face toxicity, high viscosity, and high cost of production that limit their application in FGD technology. However, to enhance their efficiency, task-specific ILs, i.e., amino acid ionic liquids (AAILs) were proposed which are environmentally friendly, easily available, biodegradable, non-toxic, and due to having two amino and carboxylic groups, they are suitable for the desulfurization process (Wang et al., 2020; Piacentini et al., 2022). An experimental investigation has shown that single-amino amino acids, especially glycine, have good absorption performance and gas saturation uptake increases to 0.461 g/g (Wang et al., 2022b). Herrera et al. (2017) confirmed by DFT computation that glycinate anion in the case of [EMIM][GLY] has a stronger interaction with captured SO_2_ (E_int_ = −126.8 kJ mol^−1^ between anion and SO_2_ in comparison to −37.0 kJ mol^−1^ between [EMIM]^+^ and gas). Yin et al. (2021) have shown that the interaction energy is directly related to the bond length and bond angle of SO_2_ and this gas capturing by silica-based porous IL is performed due to the charge transformation. Gu et al. (2013) have demonstrated that combination energy of SO_2_ and [BMIM][MeSO_4_] IL is equal to 10.86 kcal/mol and the absorption occurs due to the reducing aromaticity of the imidazolium ring and electrophilicity of SO_2_. Li et al. (2021b) have stated that carboxylic groups in the structure of IL increase absorption performance; moreover, absorption energy close to or equal to −123 kJ mol^−1^ leads to an easy release of the SO_2_ in desorption conditions.

Due to the significant potential of ILs and AAILs for SO_2_ capturing application as well as the ability to utilize computational methods to reliably simulate the molecular properties of these green and safe solvents, the important objective of the current research is to examine the physical absorption of SO_2_ in tunable imidazolium-based AAILs. The foundation for such simulations is providing a molecular understanding of the process of SO_2_ physical absorption by some imidazolium/amino acid ILs to evaluate the effect of the functional group of imidazolium cation alkyl chain on the absorption of SO_2_. Though many researchers have studied the absorption of acid gases both experimentally and theoretically, as mentioned above, the gas absorption by the AAILs is still obscure. Consequently, various structural factors affecting the absorption of SO_2_ are discussed based on the present results.

## Computational details

Density functional theory (DFT) simulations using Gaussian09 reversion A.01 (Frisch et al., 2009) were conducted to further understand the effect of the cation functional group on SO_2_ capturing by AAILs based on imidazolium cation. As DFT is one of the most efficient methods for characterizing molecular structures, conformational properties, and hydrogen bond (HB) interaction for this class of compounds, here, all computations were performed by DFT (Cao et al., 2016; Ebrahimi and Moosavi, 2018; Haddad et al., 2020). Carrying out the DFT computation leads to a precise quantification of short-range interactions (Herrera et al., 2017) between AAIL and SO_2_ gas; as a result, binding energy and favorable interaction sites will be discovered. In this line, screening and the most suitable design of ions will be performed.

All the calculations were performed using the Becke-three-parameter (B3) for the exchange part and the Lee-Yang-Parr (LYP) gradient-corrected functional with split-valence triple-zeta Pople basis set beside the polarization and dispersion functions, 6-311++G(d,p) basis set, in vacuum for the ground state optimization. Harmonic vibrational frequencies were computed at the same level to confirm that all studied geometries do not have imaginary frequency, i.e., they are corresponding to the local minima on the potential energy surfaces. Optimized structures were applied to find the binding and Gibbs Free energies, and Gaussian NBO version 3.1 (Fogarty et al., 2018) has been utilized to calculate partial atomic charges, atomic orbital occupancies, and its contribution to bonding interaction and delocalization of electron density within the SO_2_ and AAIL complexes. Determination of the atomic charges was performed at the same level of theory that was used for the geometry optimization without any symmetry constraint. Afterward, the binding energy was calculated at the same level of theory. The binding energy (BE) of AAIL-gas complexes was obtained with the following relation:

$$BE=E_{AAIL...SO_{2}}-E_{AAIL}-E_{gas}$$ (1)

where $E_{AAIL...SO_{2}}$ represents the total energy of the optimized AAIL-gas complexes and $E_{AAIL}$ and $E_{gas}$ are the total energy of the optimized isolated AAIL and gas molecule, respectively. The optimized configuration which had the lowest binding energy was selected for further investigation and discussion.

The net charge-transfer amount (NCTA) of SO_2_ absorbed by each AAIL was analyzed by the NBO population and calculated with the following relation:

$$NCTA_{SO_{2}}=C_{AAIL...SO_{2}}-C_{gas}$$ (2)

among them, $C_{AAIL...SO_{2}}$ is the charge of the gas absorbed by each AAIL and $C_{gas}$ illustrates the charge of the optimized gas molecule which is zero since it is a neutral molecule in the isolated state (free gas).

Six different initial configurations or binding sites for gas in geometry optimized AAILs were constructed, wherein the SO_2_ molecule was kept near the cation, close to the anion (both carboxylic and amine groups, separately), between the AAIL ion pairs, on the cation chain, and near hydrogen atoms of imidazolium ring sites and all these configurations were optimized. Each optimized minimum on the potential energy surface was confirmed *via* frequency analysis. Toward this end and to better understand the cation functional group effect on the interactions between the AAIL and SO_2_ from the atomic point of view, DFT computations were conducted at the same procedure to determine the BEs and NCTAs. The structures under investigation are 1-propyl-3-methylimidazolium glycinate, [C_3_MIM][GLY] besides to the cation propyl chain functionalized by five different functional groups including hydroxyl (−OH), amine (−NH_2_), carboxylic acid (−COOH), methoxy (−OCH_3_), and acetate (−CH_3_COO), represented as [C_3_OHMIM][GLY], [C_3_NH_2_MIM][GLY], [C_3_COOHMIM][GLY], [C_3_OCH_3_MIM][GLY], and [C_3_OOCCH_3_MIM][GLY], respectively; to have accessibility to task-specific AAIL for gas absorption and explore the role of cation functional group on gas absorption capacity and perform a micro-mechanism analysis. After carrying out all computations, non-covalent interaction reduced density gradient (NCI-RDG) (Johnson et al., 2010; Otero-De-La-Roza et al., 2012) by Multiwfn (Lu and Chen, 2012) as well as VMD (Humphrey et al., 1996) software and electrostatic surface potential (ESP) were computed and also applied for some visual analyses.

## Results and discussion

**FIGURE 1:**
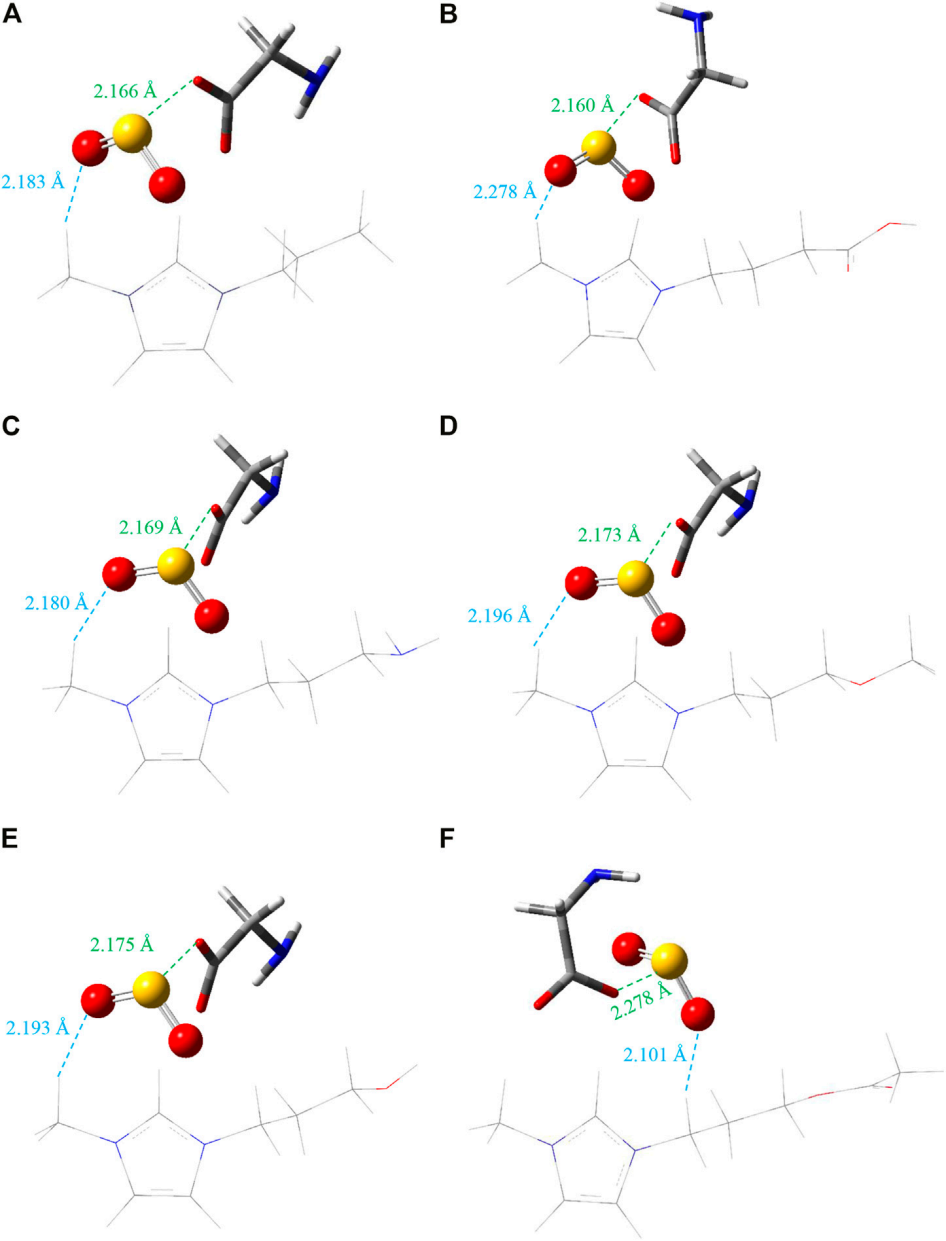
The most stable geometry for studied AAIL…SO_2_ complexes. The white, gray, blue, red, and yellow denote H, C, N, O, and S atoms, respectively. The closest distance (Å) between cation…SO_2_ and anion…SO_2_ is represented in each case. **(A)** [C_3_MIM][GLY], **(B)** [C_3_COOHMIM][GLY], **(C)** [C_3_NH_2_MIM][GLY], **(D)** [C_3_OCH_3_MIM][GLY], **(E)** [C_3_OHMIM][GLY], **(F)** [C_3_CH_3_COOMIM][GLY].

To examine the effect of the AAIL compound besides its structure on the SO_2_ capturing, which is critically important in separation performance, quantum chemistry calculations were carried out. To select the most stable structure, first of all, the cation alkyl chain length was changed from methyl to hexyl, [C_1_MIM][GLY] to [C_6_MIM][GLY], and it was found that [C_3_MIM][GLY] has the strongest interaction with SO_2_ gas. As a result, this AAIL was selected as the base of the current study. After that, six different configurations of SO_2_ concerning ion pairs, as mentioned in the previous section were optimized. To shed light on this point, the most stable configuration of captured gas with respect to AAIL was deeply studied and all the most stable structures are shown in Figure 1.

According to Figure 1, in all cases, the distance between trapped gas and anion is less than SO_2_ and cation. As the distance between the S atom of SO_2_ and O atom of the anion, S…O distance, is smaller than O…H distance, the distance between the O atom of anion and H atom of the methyl group in cation, it is understood that anion has the main role in capturing SO_2_ gas. However, in both cases, the distance is smaller than the sum of van der Waals (vdW) radii of S (1.80 Å), O (1.52 Å), and H (1.20 Å) atoms reported by Gu et al. (2013) for S…O and O…H that verifies both electrostatic and HB interactions are observed in this binding, i.e., the gas capturing is occurred due to the stronger physical interaction between anion and SO_2_ gas. In all cases, except AAIL functionalized by the acetate functional group (Figure 1F), the imidazolium ring of cation interacts with the trapped gas from its methyl side chain. In addition, the closer distance between anion and absorbed gas in comparison to cation illustrates that the SO_2_ molecule interacts more strongly with the anion; in other words, the moderate interaction causes a closer distance to the carboxylic acid group of [GLY]^−^. In a similar way to the anion, SO_2_ is an acceptor molecule here. Anion has extra electrons or negative charge; then, it plays the role of a donor species. Accordingly, SO_2_ tends to be near the anion instead of the cation and is an electrophile compound.

Distance between anion and cation before and after the absorption process is reported in Table 1. It is observable that cation-anion distance increases through gas absorption except that it does not change by absorption in [C_3_COOHMIM][GLY] AAIL. While the aforementioned distance in other systems is affected by absorption of SO_2_, the cation-anion distance of [C_3_COOHMIM][GLY] AAIL is unchanged and [C_3_CH_3_OOMIM][GLY] AAIL experiences the greatest increase which agrees well with the result of NCTA of absorbed SO_2_. There is a specific charge transfer interaction between SO_2_ and the anionic species of AAILs. The higher the anion basicity, the greater the interaction with SO_2_ and the greater the AAILs capacity for gas absorption (Mohammadi et al., 2022), which follows the same trend as cation-anion distance. It is more pronounced that E_gap_ = E_LUMO_-E_HOMO_ is the lowest one if HB interaction is formed between trapped gas and AAIL. Based on Figure 1 and Table 1, the absorbed SO_2_ gas distance is close to the cation of [C_3_CH_3_COOMIM][GLY] AAIL while it is at the uttermost distance from the anion in comparison with the other AAILs and the smallest SO_2_ absorbed NCTA has occurred; moreover, its position is different from the other target AAILs.

**TABLE 1:** Distance (Å) between anion and cation (r_cation-anion_) before and after absorption as well as SO_2_ adsorbed NCTA (e) and E_gap_ (eV).

AAIL		r_cation-anion_ (Å) before absorption	r_cation-anion_ (Å) after absorption	E_gap_ (eV)	$\Delta q_{SO_{2}}$ (e)
Without FG	[C_3_MIM][GLY]	1.84	1.89	4.73	−0.22
COOH	[C_3_COOHMIM][GLY]	1.83	1.83	4.67	−0.23
NH_2_	[C_3_NH_2_MIM][GLY]	1.77	1.90	4.74	−0.21
OCH_3_	[C_3_OCH_3_MIM][GLY]	1.83	1.89	4.74	−0.21
OH	[C_3_OHMIM][GLY]	1.84	1.91	4.73	−0.21
OOCCH_3_	[C_3_OOCCH_3_MIM][GLY]	1.64	1.95	4.30	−0.16

In general, COOH functional group increases the distance between cation and SO_2_ which may be the main reason for being the best absorbent in this study. In addition, the NCTA of SO_2_ in that system is the highest; the CH_3_COO functional group decreases SO_2_…cation distance which leads to the lower interaction energy; as can be seen, the amount of charge transfer is also the lowest value. SO_2_…anion distance is also the highest for this functional group which is the main reason for lower absorption energy; it will be discussed in the following paragraphs.

The atomic charge of the center of the atom or the atomic charge is the simplest and most intuitive concept to describe the charge distribution in a chemical system. This characteristic has important claims, such as helping to determine the state of atoms in different chemical environments, checking molecular properties, site of reaction predictions, etc. Because the atomic charge affects the dipole moment, polarizability, electronic structure, acid-base behavior, and many other molecular properties of the system, charge calculation plays an important role in the application of quantum chemical computations. Natural population analysis (NPA) is widely used for AAILs. If the charge variation in gas from the pure state to the absorbed one is small, it means that no significant charge transfer occurs. In the case of AAIL functionalized by acetate, the lowest NCTA value of SO_2_ adsorbed shows the weakest AAIL and SO_2_ interaction which leads to the lowest Wiberg bond index that enjoys strong correlations with each other (Ge et al., 2019).

**FIGURE 2:**
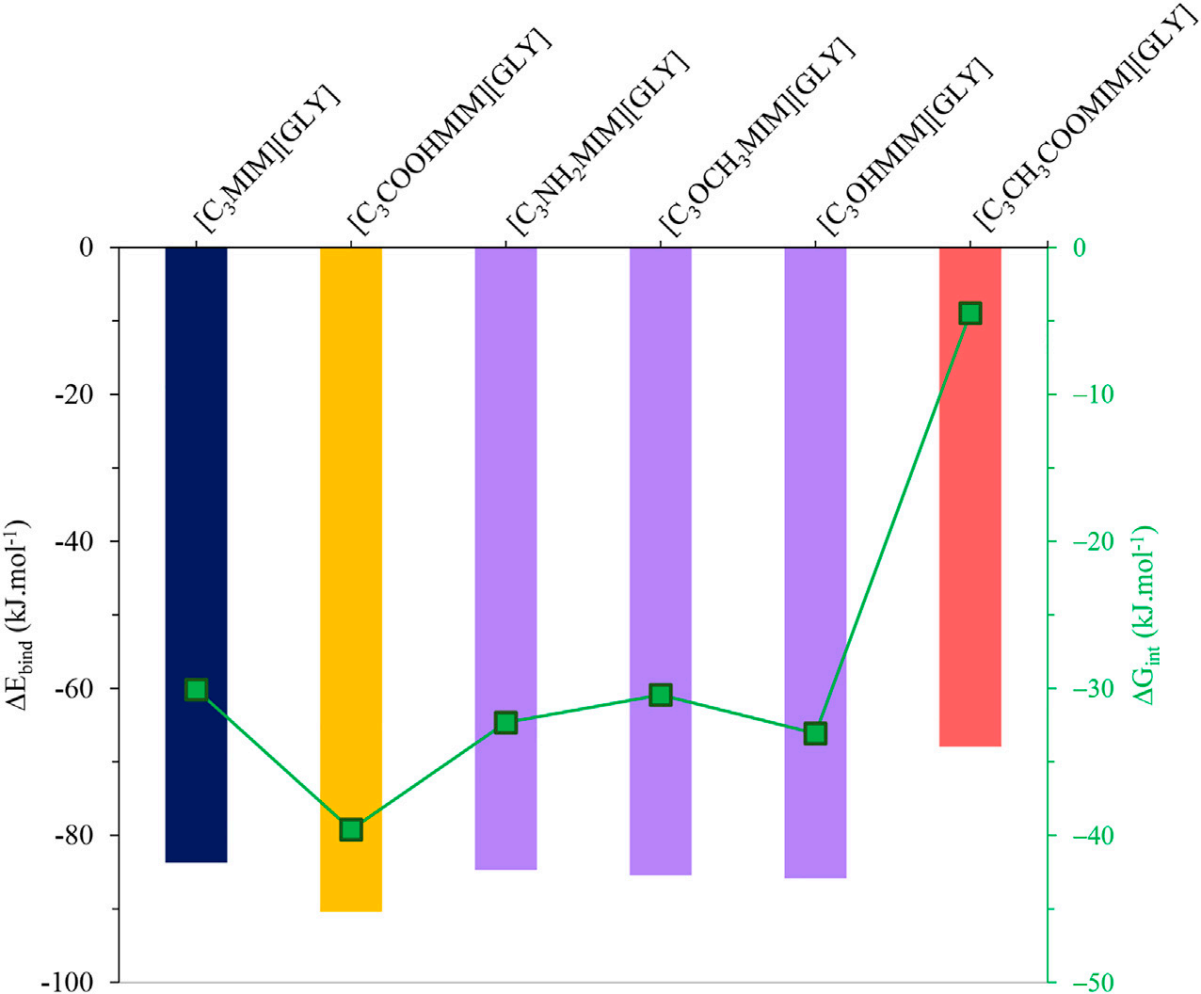
SO_2_ BE and ΔG_int_ values of absorption for the studied AAIL systems.

Interestingly, the BE and the interaction Gibbs Free energy (ΔG_int_) of all systems demonstrate quantitatively the AAIL capacity in gas absorption; see Figure 2 for more details.

As can be seen, by changing the functional group, three different regimes are observed for the studied systems: 1) COOH functional group increases the BE and ΔG_int_ of SO_2_ in AAIL, 2) CH_3_COO functional group dramatically decreases these values, and 3) inserting the functional groups of NH_2_, OCH_3_, and OH into the AAIL structure does not make an observable change in BE and ΔG_int_. Apart from the BE, the thermodynamic parameter ΔG_int_ is a negative value indicating that the absorption process is a spontaneous process. The results of ΔG_int_ also exhibit that COOH functional group intensifies the absorption while CH_3_COO decreases ΔG_int_ significantly which implies the carboxylic functional group thermodynamically improves the absorption capacity of SO_2_ by [C_3_COOHMIM][GLY] AAIL. In addition, the NH_2_, OCH_3_, and OH functional groups do not cause a substantial change in the SO_2_ capturing. Additionally, according to the BE and ΔG_int_ variations through gas absorption, it is proved that [C_3_COOHMIM][GLY] AAIL enjoys the highest stability which is in agreement with Wang et al. (2021a) results that the carboxyl group is responsible for this high amount of energy. Yin et al. (2021) have computed the interaction energy between porous ILs (PILs) and SO_2_; inspired by this work, the interaction energy is less than the values obtained in the current study. In other words, the largest interaction energy reported by Yin et al. (2021) is less than the current results showing that AAILs have more capacity in SO_2_ capturing in comparison to PILs.

**FIGURE 3:**
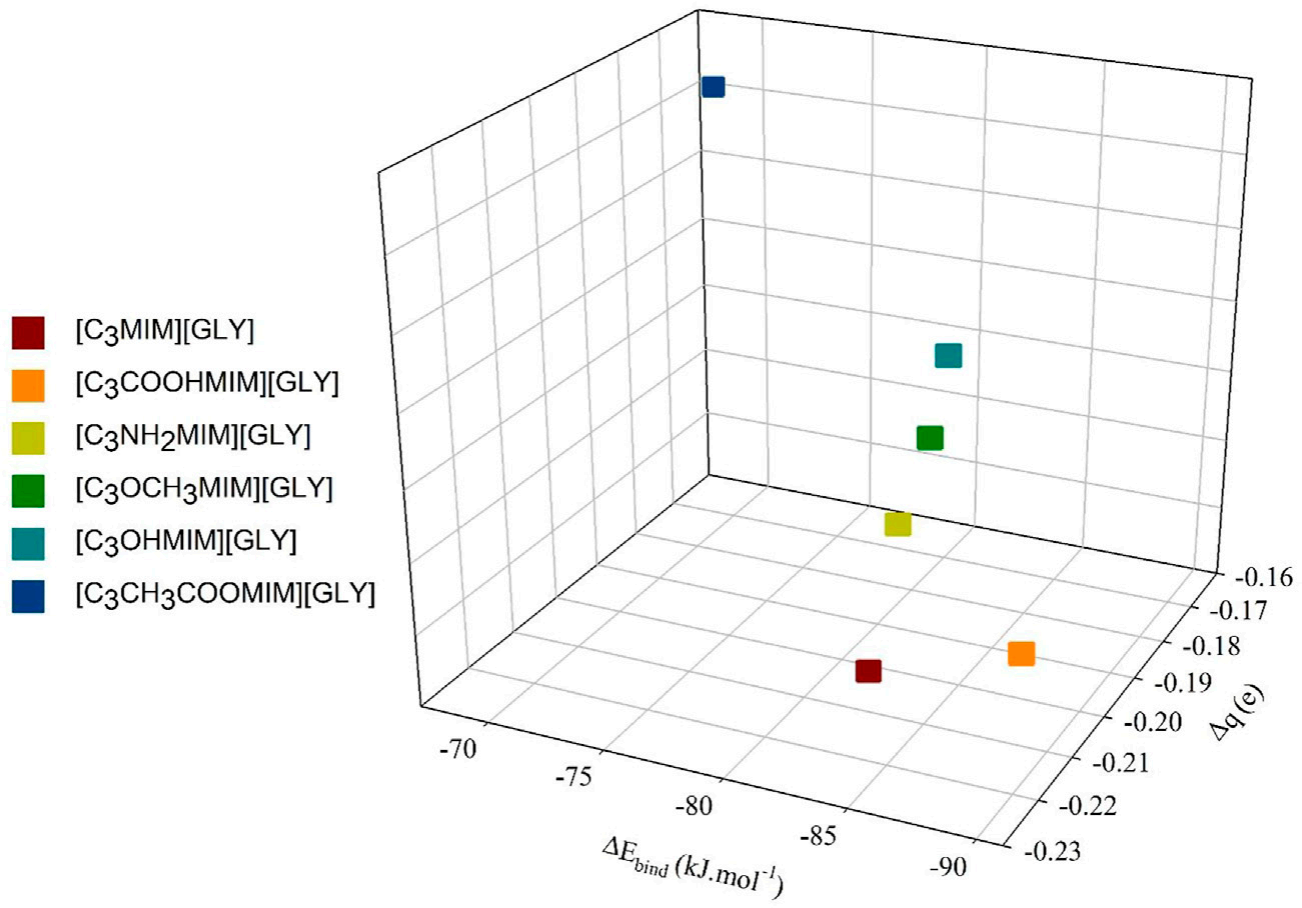
SO_2_ BE values dependency on NCTA of absorbed SO_2_ with the variation of cation functional group.

The related values of BE present quantitively the magnitudes of interaction between AAIL and SO_2_. It is noticeable that the BE values are in the range of −67.90 to −90.34 kJ mol^−1^ demonstrating a weak interaction between the absorbent and the SO_2_ gas molecule. In the case of amine, ether, and hydroxyl functional groups, the BE values are to some extent equal and the S atom of SO_2_ has also equal NCTA. AAIL with the carboxylic acid functional group is the most stable structure; it may be that SO_2_ breaks the HB between anion and cation through charge transfer of SO_2_⋅⋅⋅anion elucidating that the complex has a good ability to absorb SO_2_ gas. To evaluate charge transfer in these complexes, atomic site charges were obtained by using the NBO method in the gas phase. The charge of the SO_2_ gas after absorption is negative confirming the charge transfer from the AAIL to the gas. Based on Figure 3, there is a direct relationship between BE and $\Delta q_{SO_{2}}$ ; as a consequence, gas dissolution in the AAIL has enthalpic nature (Mondal and Balasubramanian, 2016).

The current BE values between AAIL and SO_2_ are stronger than the values obtained at the same basis set in the case of pyridinium-based ILs (Zeng et al., 2014) elucidating the higher absorption capacity of imidazolium-based AAILs functionalized the cation by ether, amine, hydroxyl, carboxylic acid, and carboxylate functional groups. The rate of change in BE due to the addition of a functional group is 33% and the overall change of the system is related to this energy, the more negative the NCTA of absorbed SO_2_, the greater the interaction between trapped SO_2_ gas and AAIL.

The structural properties of SO_2_ both in the pure state and the optimized structure of each target AAIL… SO_2_ complex are described in Table 2 which contains both S=O bond lengths (shown by *r*_S=O1_ and *r*_S=O2_ bond lengths to discriminate these changes) besides the O=S=O bond angle.

**TABLE 2:** Structure parameters (S=O bond lengths and the O=S=O bond angle) for SO_2_ absorption.

System 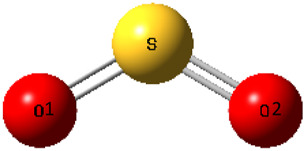	*r*_S=O1_ (Å)	*r*_S=O2_ (Å)	O1=S=O2 bond angle (°)
Pure SO_2_	1.45835	1.45834	118.69911
[C_3_MIM][GLY]+SO_2_	1.48382	1.48380	112.70670
[C_3_COOHMIM][GLY]+SO_2_	1.48229	1.48322	113.01389
[C_3_NH_2_MIM][GLY]+SO_2_	1.48379	1.48325	112.77130
[C_3_OCH_3_MIM][GLY]+SO_2_	1.48369	1.48326	112.69202
[C_3_OHMIM][GLY]+SO_2_	1.48350	1.48316	112.71120
[C_3_CH_3_COOMIM][GLY]+SO_2_	1.46948	1.47817	115.11366

According to the table, if the variation in S=O bond length and O=S=O bond angle due to the absorption is significant, it shows that the interaction between SO_2_ and AAIL is considerable. Interestingly, intramolecular parameters in gas SO_2_ are in excellent agreement with Yin et al. reported value (Yin et al., 2021). As Table 2 shows, [C_3_CH_3_COOMIM][GLY] experiences the lowest interaction between the captured gas and AAIL functionalized by the acetate functional group because of the slightest variation in SO_2_ bond parameters. In all cases, the bond length change is less than 0.5 Å which confirms a reversible physical absorption. The highest BE is related to the [C_3_COOHMIM][GLY] AAIL and the weakest one occurs in the case of [C_3_CH_3_COOMIM][GLY] AAIL; nonetheless, in both cases, the value of BE is greater than the interaction energy between SO_2_ and aqueous glycine (−47.82 kJ mol^−1^) (Wang et al., 2021b) and [C_4_MIM][MeSO_4_] (−45.438 kJ mol^−1^) (Gu et al., 2013), respectively. Considering these facts, if the BE value of AAIL…SO_2_ under environmental conditions is appreciated, the AAIL can capture the gas in harsh conditions. In addition, the target AAILs can release the captured gas with physical variations such as temperature or pressure change. As the characteristics of [C_3_NH_2_MIM][GLY], [C_3_OCH_3_MIM][GLY], and [C_3_OHMIM][GLY] AAILs are similar to each other, in the next paragraphs, it is only concentrated on [C_3_MIM][GLY], [C_3_COOHMIM][GLY], [C_3_CH_3_COOMIM][GLY] AAILs complexed with SO_2_.

**FIGURE 4:**
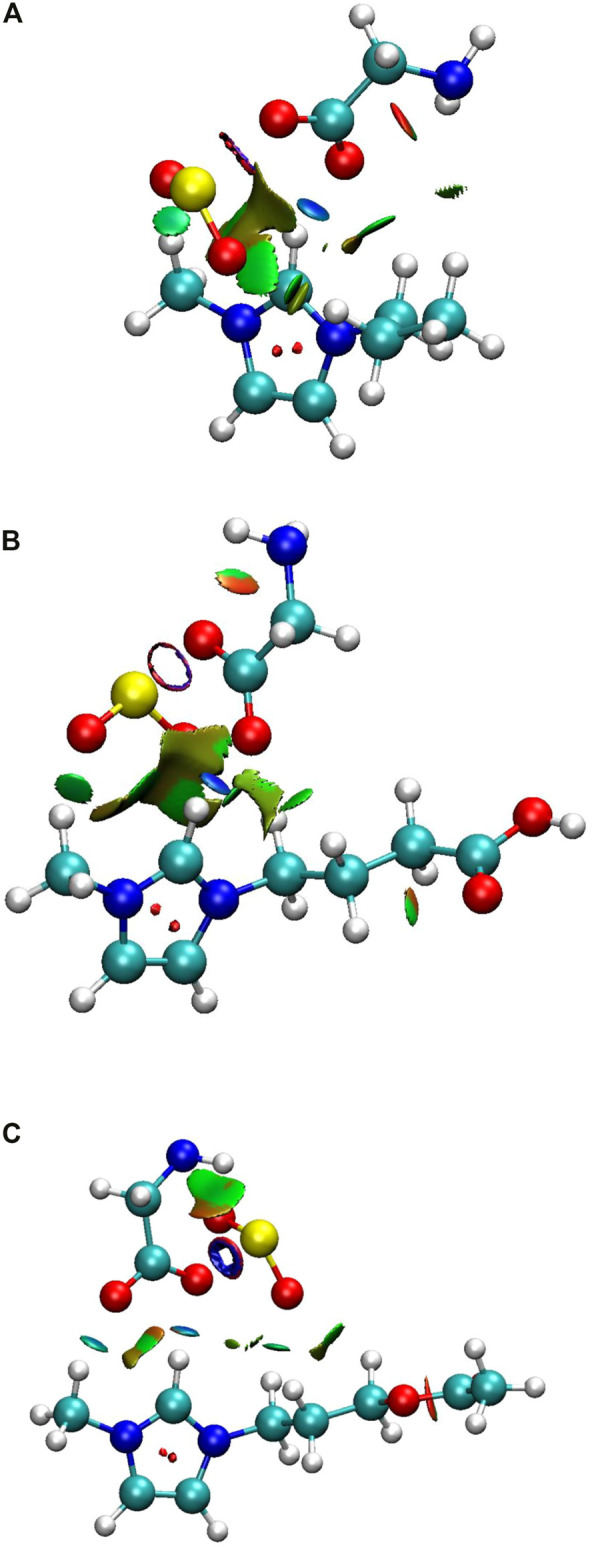
NCI-RDG analyses of AAIL complexed with SO_2_ **(A)** [C_3_MIM][GLY], **(B)** [C_3_COOHMIM][GLY], and **(C)** [C_3_OOCCH_3_MIM][GLY].

NCI-RDG analyses were performed for each AAIL…SO_2_ complex to take into consideration the nature of non-covalent interactions. Furthermore, the goal of NCI evaluation is to find the weak HB, vdW, and steric effect interaction domains from wave-function calculations. This analysis represents a three-dimensional (3D) scheme of interactions in 3D space. Figures 4A–C display the color-filled isosurfaces of the interactions of pristine AAIL, [C_3_MIM][GLY], and ones functionalized by carboxylic acid and acetate, i.e., [C_3_COOHMIM][GLY] and [C_3_CH_3_COOMIM][GLY] AAILs, with trapped SO_2_. If the colored-filled isosurface is between AAIL and SO_2_, gas capture has occurred. Whenever this domain is green, there is a vdW interaction, blue domain shows an HB interaction which is present in [C_3_MIM][GLY] and [C_3_COOHMIM][GLY] AAILs; in general, the red shows repulsive interactions. According to the figures, blue isosurfaces in the region between the S atom of SO_2_ and the O atom of the anion point to considerable electrostatic interactions. Captured SO_2_ gas is near to the methyl group of the cation if there is a vdW interaction between gas and cation that is in agreement with Li et al. (2021a) results. It is observable that the interaction between anion and SO_2_ for the AAIL functionalized by COOH is the strongest in comparison with two other AAILs ([C_3_MIM][GLY] and [C_3_COOHMIM][GLY]). In addition, the S atom of SO_2_ orients towards the anion, and the two O atoms of SO_2_ rotate toward the cation in [C_3_MIM][GLY] and [C_3_COOHMIM][GLY] AAILs (Figures 4A, B). While only one of the O atoms of SO_2_ gas rotates towards the cation, the other orients toward the anion (Figure 4C). This orientation may cause weaker interaction between SO_2_ and absorbent. As can be seen, COOH functional group improves SO_2_/AAIL interaction because of the strong interaction of the S atom with the anion and the O atoms with the cation. Consequently, these observations confirm the considerable sensitivity of AAIL factionalized by COOH to SO_2_ gas in comparison to the other target AAILs.

**FIGURE 5:**
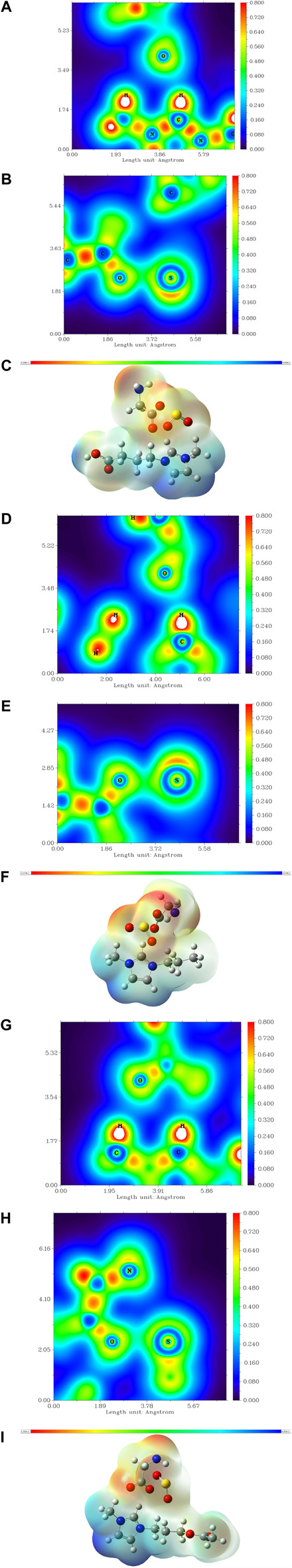
LOL and ESP analyses of the interaction between the AAILs and SO_2_: **(A)** LOL of anion and cation in [C_3_MIM][GLY]…SO_2_ complex, **(B)** LOL of SO_2_ and anion of [C_3_MIM][GLY]…SO_2_ complex, **(C)** ESP of [C_3_MIM][GLY]…SO_2_ complex, **(D)** LOL of anion and cation of [C_3_COOHMIM][GLY]…SO_2_ complex, **(E)** LOL of SO_2_ and anion of [C_3_COOHMIM][GLY]…SO_2_ complex, **(F)** ESP of [C_3_COOHMIM][GLY]…SO_2_ complex, **(G)** LOL of anion and cation of [C_3_OOCCH_3_MIM][GLY]…SO_2_ complex, **(H)** LOL of SO_2_ and anion of [C_3_OOCCH_3_MIM][GLY]…SO_2_ complex, **(I)** ESP of [C_3_OOCCH_3_MIM][GLY]…SO_2_ complex.

Figure 5 exhibits the localized orbital locator (LOL) and ESP analysis of the interaction between each AAIL and SO_2_ studied in the gas phase at B3LYP/6-311++G(d,p) level of theory. Normally, a great value of the LOL points to the covalent bond and a small value indicates the electrostatic interactions (Nkungli and Ghogomu, 2017).

According to Figure 5, LOL values are insignificant confirming an electrostatic interaction between the AAILs and the SO_2_. In addition, ESP analysis is a representation of the most stable configuration and a guide for molecular structure optimization. It can be applied to detect the reactive sites of a molecule in the systems (Politzer and Murray, 2002). The values of the ESP on the 3D map surface follow the trend of red < orange < yellow < green < blue. The blue regions depict electron deficiency (nucleophilic reactivity) and the red regions point to the relative abundance of electrons (electrophilic reactivity). Consistent with Figure 5C, SO_2_ is a reactive nucleophilic center for coordination with [C_3_MIM][GLY] AAIL and ESP around SO_2_ in the [C_3_COOHMIM][GLY] AAIL (Figure 5F) is green color meaning that the electron density is balanced. However, in the case of AAIL functionalized by CH_3_COO, the electron density is somewhat out of equilibrium (Figure 5I). Studying the non-covalent interactions including vdW, HB, and electrostatic is of great importance and can be also carried out by ESP analysis which is suitable for a qualify interaction analysis. HB is the main factor in gas absorption by AAIL. The results demonstrate that the positive charge on the S atom (around 1.60 e) of SO_2_ and the negative charge of the O atom from glycinate anion (about −0.76 e) lead to interaction between AAIL and SO_2_. When SO_2_ interacts with AAIL, in agreement with Yin et al. (2021) study, SO_2_ interaction with cation from O atom by HB occurs and its interaction with the anion from S atom belongs to electrostatic interaction. As a result, it seems that increasing the number of carboxylate groups in AAIL structure is an efficient parameter for SO_2_ absorption by AAIL.

**FIGURE 6:**
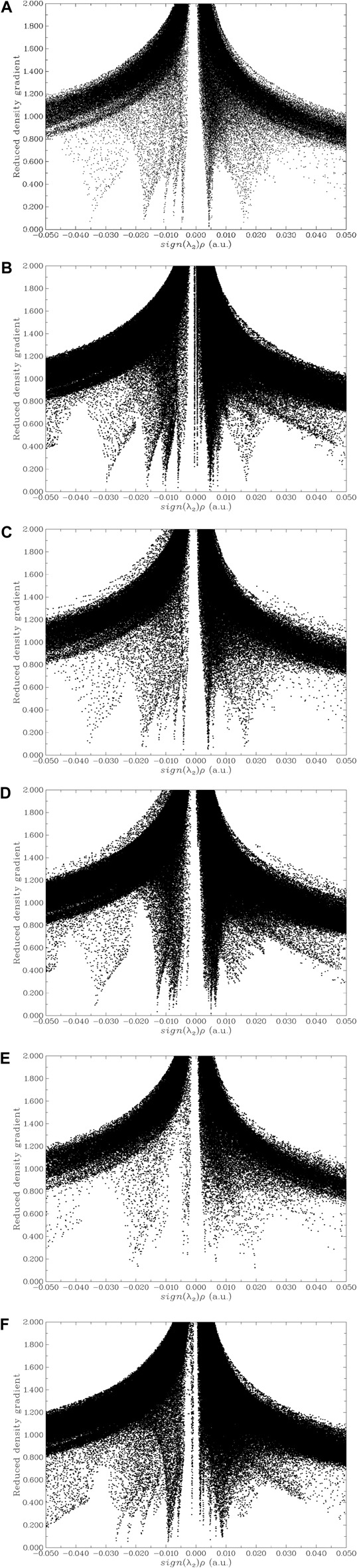
The non-covalent interaction (NCI) analyses for studied AAILs and AAIL…SO_2_ complexes. **(A)** [C_3_MIM][GLY] AAIL, **(B)** [C_3_MIM][GLY]…SO_2_, **(C)** [C_3_COOHMIM][GLY], **(D)** [C_3_COOHMIM][GLY]…SO_2_, **(E)** [C_3_CH_3_COOMIM][GLY], and **(F)** [C_3_CH_3_COOMIM][GLY]…SO_2_.

The non-covalent interaction (NCI) analysis can be used to determine the interactions based on electron density and the sign of the second derivative in the perpendicular direction of the bond (λ_2_) (Geng et al., 2022). This analysis provides a clear description of the attractive and repulsive interactions between AAILs and SO_2_. A large positive value of sign (*λ*_2_ *ρ*) points to steric repulsion (Figure 6), a large negative value of sign (*λ*_2_ *ρ*) refers to the HB (Figure 6), and the value near to zero (λ_2_ ≤ 0) denotes the vdW interactions (Figure 6). By comparing the NCI of these three systems, it sheds light on the interaction types and the spatial positions between AAIL and SO_2_ at the atomic and molecular levels; it is observable that the COOH functional group decreases the steric effect after absorption of SO_2_ while the CH_3_COO functional group significantly increases the steric effect after absorption. COOH functional group does not change HB and vdW interactions whereas the CH_3_COO functional group shrinkages this interaction. It is worth mentioning that before absorption of SO_2_ the steric effects of all systems are similar and absorption of SO_2_ significantly decreases the steric effect; in all systems, the vdW and HB experience a slight decrease after absorption. The absorption mechanism systematically investigated from the quantum chemical point of view shows that RDG analysis is a clear visualization method for weak interaction sites. It is crystal clear that vdW interaction between [C_3_CH_3_COOMIM][GLY] AAIL and SO_2_ is not absent and the weakest is present while the strongest vdW interaction is seen in [C_3_COOHMIM][GLY]…SO_2_ complex system; therefore, [C_3_COOHMIM][GLY] AAIL has the ability to inhibit the interaction of SO_2_ with other gases and improve its absorption rate.

The above facts designate that weak interaction magnitude is related to BE but the interaction can not be observed graphically and tangible. Therefore, Johnson et al. (2010) have suggested RDG analysis method that can display the non-covalent interaction graphically, Figure 6. According to these figures, a spike near zero and the right side is a non-covalent bond. A λ_2_ > 0 is a sign of non-bonding interaction and λ_2_ < 0 shows a bonding interaction, where λ_2_ is the eigenvalue of the electron density (Hessian) second derivative matrix. The multiplication of electron density and λ_2_ has a value with a sign that shows the interaction type and its intensity. A value of $\rho sign\left(\lambda_{2}\right)<-0.02$ means a strong interaction (HB, halogen bond, etc.), if $-0.01<\rho sign\left(\lambda_{2}\right)<+0.01$ , it shows a vdW non-covalent interaction, and $\rho sign\left(\lambda_{2}\right)>0.01$ is a mutual exclusion such as potential resistance effect in a ring and cage, i.e., strong non-bonding overlap.

**FIGURE 7:**
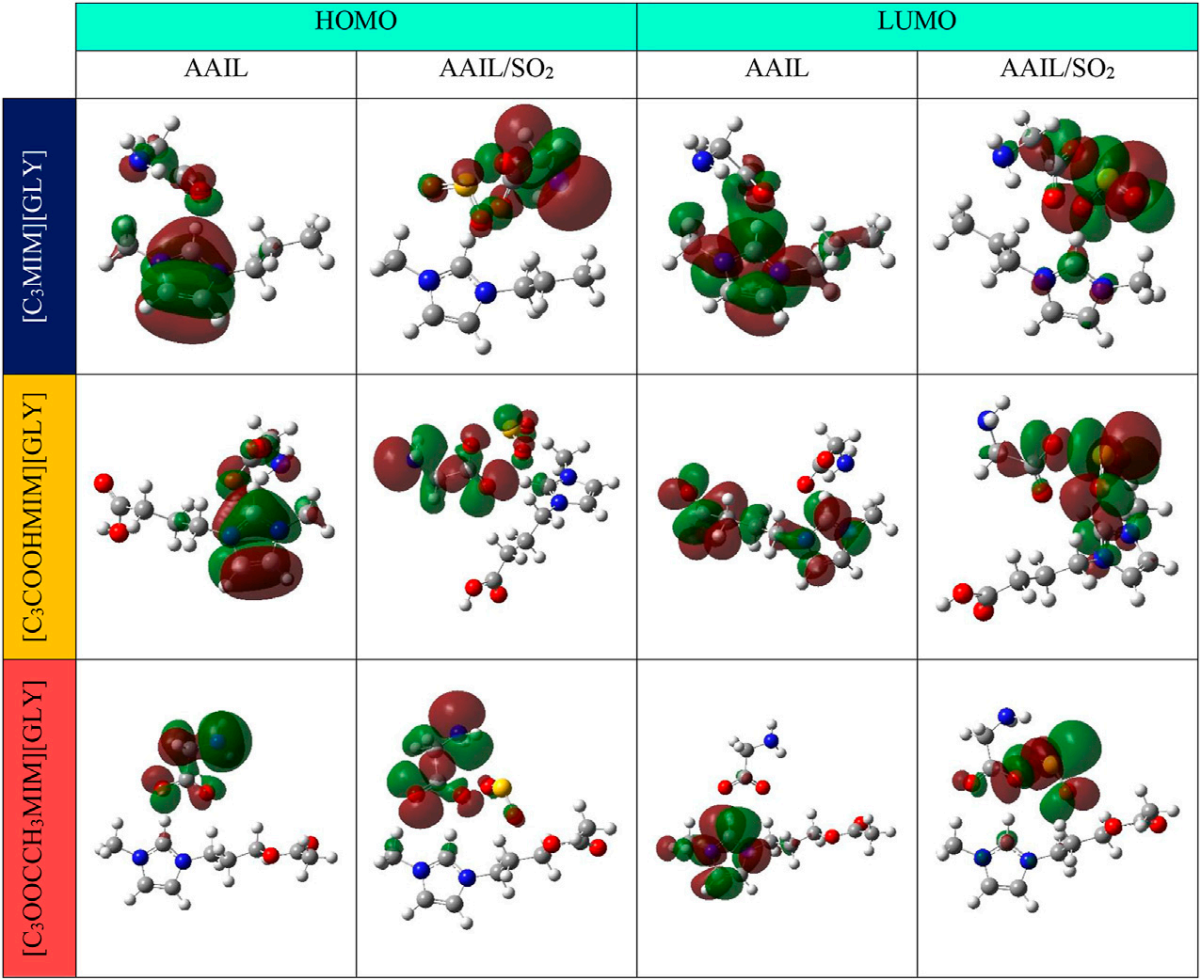
Frontier molecular orbitals for pure AAILs and AAIL…SO_2_ complexes.

To get a better insight into the absorption of SO_2_, the Frontier Molecular Orbital (FMO) analysis was also performed and is plotted in Figure 7. HOMO is a bond orbital or lone pair while LUMO is an anti-bond orbital. Based on Fukui’s FMO theorem, whenever HOMO or other filled orbitals are near LUMO or other unoccupied orbitals, electron exchange from HOMO to LUMO of the other molecule is easier and the attraction is strong. For a pure AAIL (which is shown by AAIL in Figure 7) HOMO of [C_3_MIM][GLY] and [C_3_COOHMIM][GLY] AAILs is composed of a balanced distribution of the orbitals on the anion and ring of the cation. Whereas, HOMO of [C_3_CH_3_COOMIM][GLY] is distributed on the anion. After adding the SO_2_ (which is shown by AAIL/SO_2_ in Figure 7), the orbitals are concentrated on anion in all systems. Meanwhile, the orbital of SO_2_ shows a slightly bigger overlap with the AAIL functionalized by the COOH group. While it shows the lowest overlap with AAIL functionalized by the acetate group. 2p atomic orbitals of C, O, and N formed the highest occupied molecular orbital (HOMO) of the AAILs which is delocalized over the SO_2_ gas. Furthermore, the LUMO of the system involving [C_3_COOHMIM][GLY] AAIL and SO_2_ shows an overlap between the cation, anion, and SO_2_ while the other system shows anion and SO_2_ overlap. All these observations point out the favorability of the COOH functional group for the absorption of SO_2_. Therefore, RDG besides ESP designate that HB and electrostatic interactions of O…H and S…O, respectively, play the role in the absorption gas process. The electron transform from HOMO of anion to LUMO of SO_2_ arises throughout the gas capturing. As a result, S…O interaction is a π-hole interaction since the V-shaped SO_2_ molecule with a π delocalized bond has an interaction with lone pair electrons of the O atom in the carboxylic group of the anion. This interaction appropriately matches with π-hole bonding interaction (Zhu et al., 2021).

**FIGURE 8:**
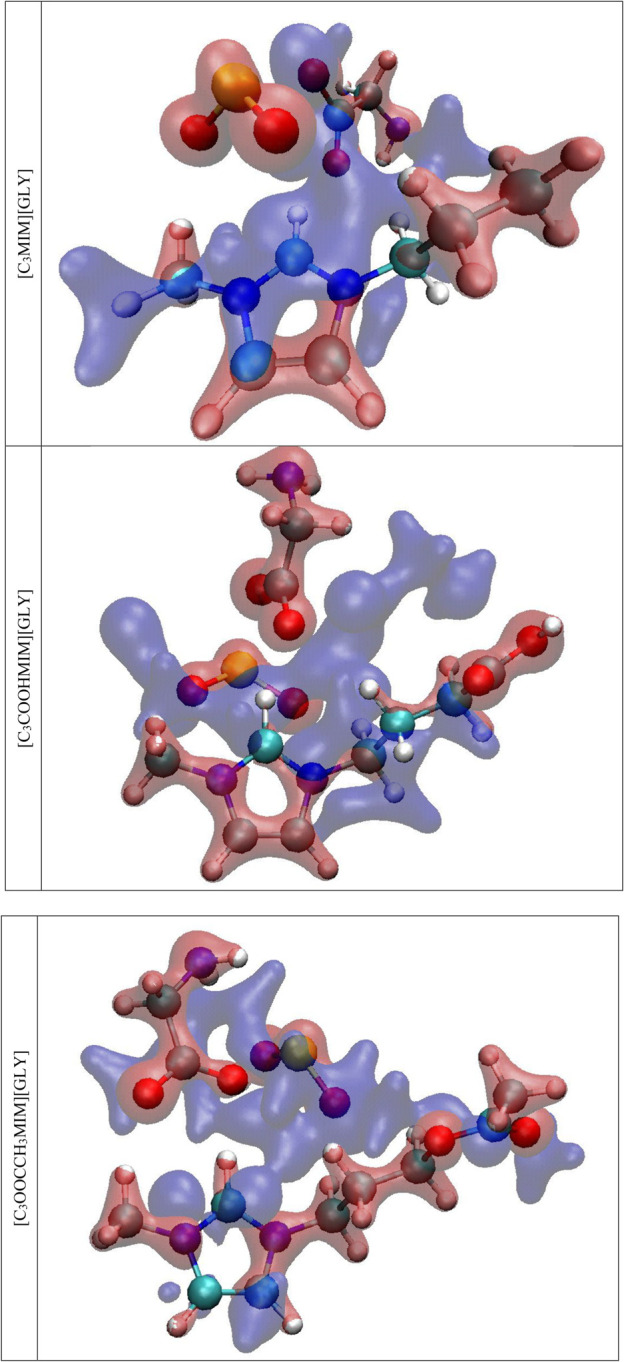
EDD for the complex of AAIL…SO_2_.

The isosurface of electron densities is the regions with increasing and decreasing electron density after SO_2_ accommodation in AAIL with an isovalue of (−0.15 and 0.15 a.u.). These regions with an increase in density are shown in red and a decrease in electron density, electron density difference (EDD), is shown in blue in Figure 8.

As the figure illustrates, SO_2_ experiences an enhancement in electron density. Wherever the distance is minimum, the EDD is the maximum, and SO_2_ absorbed NCTA is also confirmed. Additionally, the anion charge depletion is greater than the cation which verifies the anion’s critical role in gas absorption. In summary, the solubility of SO_2_ in AAILs with different functionalities demonstrates that electron-withdrawing groups such as carboxylic acid reduce the chemical absorption enthalpy as well the reconstruction of electricity consumption will be disrated.

## Conclusion

Conventional ILs face economic issues, toxicity, and poor biodegradability; consequently, it is pivotal to replace them with task-specific ion pairs and make them more suitable for acidic gas capturing. The synergistic effect of cation functionalized by electron-donating groups for SO_2_ absorption in AAILs based on imidazolium cation was under consideration. For this purpose, the glycine amino acid played role as AAIL anion and effect of the functional groups on cation with propyl alkyl chain length functionalized by hydroxyl (OH), amine (NH_2_), carboxyl (COOH), methoxy (OCH_3_), and acetate (CH_3_COO) was under evaluation. In order to intuitively understand the magnitude of the force more, the binding energy (BE), the captured gas distance concerning the cation and anion, and SO_2_ structure change were calculated. It was made clear that the carboxylic acid functional group has a great contribution in the absorption of SO_2_ by AAIL while CH_3_COO dramatically decreases, and adding the functional group of NH_2_, OCH_3_, and OH does not affect the absorption energy of SO_2_ in the target AAILs. The Gibbs free energy of SO_2_ absorption shows that the AAIL functionalized by the carboxylic acid group (COOH) is a thermodynamically favorable solvent. COOH functional group decreases the distance between anion and SO_2_ which may be the main reason for being the best absorbent. In addition, the number of charge transfers of SO_2_ in that system was the highest. Non-covalent interaction analysis investigates the nature of interactions. Comparing the NCI demonstrates that the COOH functional group decreases the steric effect. However, the CH_3_COO functional group significantly increases the steric effect after absorption. To distinguish the weak interaction between AAIL and captured gas, the RDG map was used; the acetate functional group make diminutions in HB interaction followed by reduction in NCI-RDG, LOL, ESP, EDD, and HOMO-LUMO results.

## Data Availability

The original contributions presented in the study are included in the article/Supplementary Material, further inquiries can be directed to the corresponding author.
